# Green sonochemical synthesis of BaDy_2_NiO_5_/Dy_2_O_3_ and BaDy_2_NiO_5_/NiO nanocomposites in the presence of core almond as a capping agent and their application as photocatalysts for the removal of organic dyes in water

**DOI:** 10.1039/d0ra10288a

**Published:** 2021-03-19

**Authors:** Seyede Raheleh Yousefi, Azam Sobhani, Hassan Abbas Alshamsi, Masoud Salavati-Niasari

**Affiliations:** Institute of Nano Science and Nano Technology, University of Kashan Kashan P. O. Box. 87317-51167 Islamic Republic of Iran Salavati@Kashanu.ac.ir +98 31 55913201 +98 31 55912383; Department of Chemistry, Kosar University of Bojnord Bojnord Islamic Republic of Iran Sobhani@kub.ac.ir; Department of Chemistry, College of Education, University of Al-Qadisiyah Diwaniya 1753 Iraq

## Abstract

The present work reports the sonochemical synthesis of DBNO NC (dysprosium nickelate nanocomposite) using metal nitrates and core almond as a capping agent. In addition, the effects of the power of ultrasound irradiation were investigated. The BaDy_2_NiO_5_/Dy_2_O_3_ and BaDy_2_NiO_5_/NiO nanocomposites were synthesized with sonication powers of 50 and 30 W, respectively. The agglomerated nanoparticles were obtained using different sonication powers, including 15, 30, and 50 W. The results showed that upon increasing the sonication power, the particle size decreased. After characterization, the optical, electrical, magnetic, and photocatalytic properties of the NC were studied. The nanocomposites showed an antiferromagnetic behavior. In this study, the photocatalytic degradations of two dyes, AR14 and AB92, were investigated in the presence of DBNO NC. Furthermore, the effects of the amount of photocatalyst, the concentration of the dye solution, the type of organic dye, and light irradiation on the photocatalytic activity of the nanocomposite were studied. The results showed that with an increasing amount of catalyst and decreasing concentration of dye, the photocatalytic activity of the nanocomposite was increased. This activity for the degradation of AR14 is higher than that of AB92. Both AR14 and AB92 dyes show higher photocatalytic degradation under UV irradiation than under Vis irradiation.

## Abstract 2

The sonochemical synthesis of Dy_2_BaNiO_5_ nanocomposites is reported, for the first time. The metal nitrates and core almond are used as precursors and capping agent, respectively. Effect of sonication power is investigated in this work.

## Graphical abstract

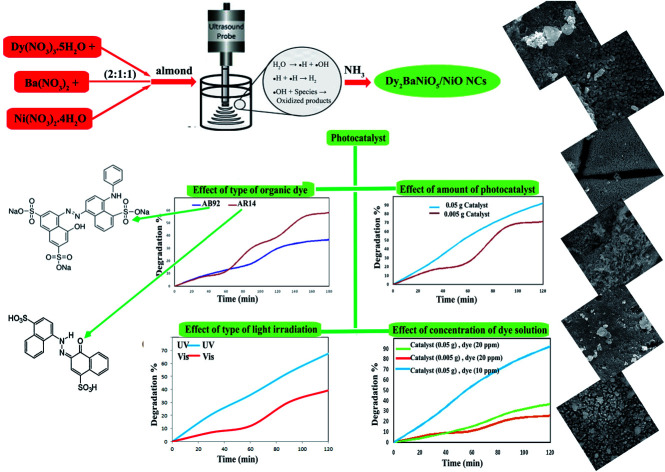

## 1. Introductions

The rare-earth nickelates, R_2_BaNiO_5_, (R = Pr–Gd, Dy, Tm, and Y) with an orthorhombic crystal system are crystallized in the *Immm* space group.^1,2^ These nickelates have attracted a considerable amount of attention.^3–5^ They have one-dimensional chains of vertex-sharing NiO_6_ flattened octahedra along the *a*-axis.^6^ The chains are only linked through Ba^2+^ and Dy^3+^ ions; there is no direct oxygen bond between them. Owing to this peculiar structure, they show one dimensional and interesting magnetic properties.^7^ The Ni–O–R–O–Ni superexchanges can cause the interaction between the chains.^8^ The dysprosium nickelate (DBNO) synthesized in this work belongs to this family of rare-earth nickelates. The detailed structure of the nickelates has been studied by Garcia-Matres.^2^ The presence of the one-dimensional structures, along with a strongly anisotropic crystal structure, has resulted in the nickelates attracting a large amount of attention. Some of the nickelates have two structures: the Ni ions can be in an octahedral or pyramidal coordination. These coordination structures are related to the synthesis conditions.^6,9^

Owing to the Dy-mediated interaction between the nickel chains with *S* = 1, DBNO has an antiferromagnetic (AFM) ordering at TN = 59 K.^10^ Perfectly straight Ni–O–Ni chains with a strong superexchange interaction (*J*_‖_ ∼ 25 meV) are directed along the crystallographic *a*-axis, and Ni^2+^ ions carrying spin *S* = 1 form Haldane chains well separated one from another. In compounds with magnetic rare earth ions, *J*_⊥_ is weak compared to *J*_‖_(*J*_‖_/*J*_‖_ ∼ 10^−2^), but is large enough to establish AFM ordering at low temperatures.^8^ In this study, DBNO was used as a model compound for studying Haldane magnetism,^11,12^ as these nickelates show a multiferroic behavior.^5^

Galkin *et al.* studied the temperature behavior of the crystal-field levels of the Dy^3+^ ion in DBNO. They used optical spectroscopy for this purpose.^13^ Singh *et al.* investigated the magnetic properties of Dy_2_BaNiO_5_,^5^ and reported subtle and broad magnetic anomalies for this nickelate at approximately 10 K and also 30–50 K.^5^ Moessbauer spectroscopy has also been used for investigation of the magnetic structure of the nickelates.^14^ Klimin *et al.* investigated the optical properties of the nickelates, they also used magnetic studies reported in the literature, and suggested magnetic structures for some nickelates.^8^

Garcia-Matres *et al.* investigated the magnetic behavior of the R_2_BaNiO_5_ by neutron diffraction and magnetization measurements.^7^ Klimin *et al.* synthesized a series of polycrystalline R_2_BaNiO_5_ (R = Nd, Sm, Eu, Gd, Dy, Ho, Er, and Tm) using the solid-state reaction method.^8^ They used Er_2_O_3_, Y_2_O_3_, NiO, and BaCO_3_ as precursors. The rare-earth nickelates were characterized using XRD. Klimin *et al.* used the spectroscopic method to study the magnetic ordering and determined the type of magnetic structure for the nickelates in the magnetically ordered state.^8^ Basu *et al.* investigated the magnetic properties of Er_2_BaNiO_5_.^15^ This nickelate has an antiferromagnetic order below 32 K. Basu *et al.* showed that these nickelates are prone to multiferroicity under favorable circumstances owing to the displacive-type mechanism that distorts the O6 octahedra, thereby lifting the point of inversion symmetry.^15^ Basu *et al.* also investigated the magnetic behavior of the Dy_2_BaNiO_5_.^16^ Galkin *et al.* studied optical transmission spectroscopy of the Dy_2_BaNiO_5_, the spectroscopy was performed in the region of the f–f transitions of the Dy^3+^ ion at 5–300 K.^13^ Alvarez and Valenti investigated the magnetic properties of R_2_BaNiO_5_ and showed the coexistence of a three-dimensional magnetic long-range order with one-dimensional quantum gap excitations.^17^ Upadhyay *et al.* studied the magnetic transitions of Tb_2_BaNiO_5_.^18^ The Tb_2_BaNiO_5_ has an AFM order below the Neel temperature (TN = 63 K). In addition, Popova *et al.* studied the magnetic structure and interchain interactions in the mixed-spin nickelate containing two different rare earth metals, (Er_0.25_Gd_0.75_)_2_BaNiO_5_, using optical spectroscopy.^19^ They also determined the low-temperature magnetic properties of Nd_2_BaNiO_5_ by exchange splitting of the ground state^20^ and measured the diffuse transmittance spectra of this nickelate. Furthermore, the optical spectroscopy of Nd_2_BaNiO_5_ and Nd_0.1_Y_1.9_BaNiO_5_ were investigated by this group.^21^ The magnetic studies of R_2_BaNiO_5_ were also continued by Nénert and Palstra.^22^ They studied the interplay between the magnetic and dielectric properties of Ho_2_BaNiO_5_ on a polycrystalline sample and revealed the linear magnetoelectric effect in this nickelate.^22^ Narozhnyy *et al.* also investigated the absorption spectra of the mixed chain nickelates.^23^ In previous works, the photocatalytic properties of rare-earth nickelates have been not studied. Thus, in this work investigation of these properties has been emphasized, as these have been absent in previous studies.

The presence of a large number of organic pollutants in water pose a serious water pollution threat to human society.^24^ These pollutants can affect human health and include endocrine-disrupting chemicals and antibiotics.^25^ A variety of pollutant treatment methods have been investigated by researchers, to protect the environment. Photocatalysis, as an advanced oxidation technology, is easy to operate and environmentally friendly.^26^ Photocatalysis can be used to decompose various substances by oxidation and reduction reactions induced by the migration of h^+^ and e^−^ to the surface of the photocatalyst^27–29^ and is an attractive approach to solving environmental problems.

Using solar energy for the removal of pollutants, H_2_ evolution by water splitting, and photoreduction of CO_2_ to synthesize carbon-bearing fuels is a green technique.^30–33^ TiO_2_ photocatalysts can only be used under UV light.^34^ Thus, developing a photocatalyst with a reasonable photocatalytic performance under both UV and Vis light has attracted considerable attention. Thus, the main purpose of this article is to synthesize photocatalysts and investigate their photocatalytic activity for the degradation of organic dyes under both UV and Vis light.

## 2. Experimental

### 2.1. Materials and experiments

All of the materials used in this work, Dy(NO_3_)_3_·5H_2_O, Ba(NO_3_)_2_, Ni(NO_3_)_2_·4H_2_O, and NH_3_ (25%) were purchased from Merck company. The ultrasonic irradiation was performed using a Sonicator 3000 (Bandeline, MS 72, Germany). This generator was multi-wave and its titanium probe was a converter/transducer with a 12.5 mm diameter. The sonicator included a microtip probe, power generator, and piezoelectric lead zirconate titanate crystal. Isopropyl alcohol was used throughout to clean the tip.^35^ The probe was immersed 1 cm below the surface of the liquid. The ultrasound instrument worked at 20 kHz and its maximum power was 60 W. XRD patterns were obtained using Ni-filtered Cu Kα radiation of an X-ray diffractometer (Philips X'pertPro, *λ* = 1.54 Å). A Philips microscope (XL30) was used for energy-dispersive X-ray spectroscopy (EDS) analysis. The morphologies of the products were visualized using scanning electron microscopy (SEM) (TESCAN Mira3 FE-SEM) and transmission electron microscopy (TEM) (Philips EM208). The accelerating voltage used to obtain the TEM images was 200 kV. A Fourier transform infrared spectroscopy (FT-IR) spectrum was taken using a spectrophotometer (Shimadzu FTIR-4300) with KBr pellets. The high resolution transmission electron microscopy (HRTEM) images were taken using a JEM-2100 with an accelerating voltage of 200 kV. The diffuse reflectance spectroscopy (DRS) spectrum of the nanocomposite was studied using an Ava Spec-2048TEC spectrometer. A cyclic voltammetry (CV) curve was obtained using a SAMA 500 potentiostat in Isfahan, Iran. The magnetic properties of the products were studied using a vibrating sample magnetometer (VSM), Meghnatis Kavir Kashan Co., in Kashan (Iran).

### 2.2. Synthesis of DBNO NC

**Scheme 1:**
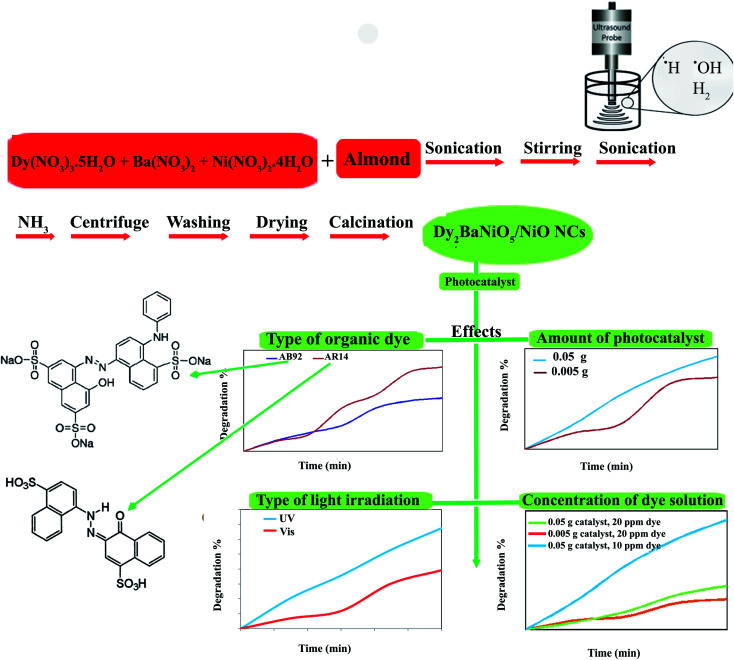
Schematic diagram illustrating the synthetic route and investigation of the effects of different agents in the photocatalytic degradation of AB92 and AR14.

The Dy_2_BaNiO_5_ nanocomposite (DBNO NC) was synthesized using an ultrasonic route at atmospheric pressure, and then calcined at 800 °C for 2 h. First, the core almond was treated using ultrasound in 10 ml of deionized water for 20 min. Then, an aqueous solution of Dy (0.50 g), Ba (0.15 g), and Ni (0.10 g) nitrates with a 2 : 1 : 1 molar ratio was prepared. The almond solution was added to the metal nitrates solution. The prepared solution was stirred for 10 min and then sonicated. The NH_3_ solution was added into the final solution for 20 min until the pH value reached 8.0 (Scheme 1). The precipitates were centrifuged, washed using distilled water, dried at 70 °C for 12 h, and then calcined at 800 °C for 2 h. In this study, the effect of the sonication power was investigated (Table 1). The core almond is used as a capping agent, it acts as a stabilizing agent and provides colloidal stability as well as preventing agglomeration and stopping uncontrolled growth. The final morphology of the products depends on the capping agent, which is adsorbed on the surface. The capping agents are the key to obtaining small-sized nanoparticles and are very frequently used in the colloidal synthesis of nanoparticles to avoid overgrowth.

**Table 1:** Reaction conditions for the sonochemical synthesis of DBNO NC in this work

Sample	Capping agent	Solvent	Sonication time (min)	Sonication power (W)	Calcination
1	Core almond	H_2_O	20	15	800 °C/2 h
2	Core almond	H_2_O	20	30	800 °C/2 h
3	Core almond	H_2_O	20	50	800 °C/2 h

### 2.3. Photocatalytic measurements

Two anionic dyes, acid red 14 (AR14) and acid blue 92 (AB92) were selected for investigation of the photocatalytic activity of the as-synthesized DBNO NC. The photocatalytic efficiency of NC was investigated under both UV (Osram, 400 W) and Vis (fluorescent lamp) irradiation. In this study, the dye solution and photocatalyst, with different concentrations and amounts, were utilized in a glass reactor. Two solutions of dyes with 10 and 20 ppm concentrations and two amounts of photocatalysts, including 0.05 and 0.005 g were prepared. A quartz reactor was used for performing the reactions. The catalyst was added into the dye solution, suspended, and stirred in the darkness. After aerating in darkness to achieve the adsorption–desorption equilibrium (for 30 min), the mixture was irradiated using UV and Vis irradiation. The temperature of the mixture was maintained at room temperature. The percentage of degradation was calculated as followed: *D*% = [(*A*_0_ − *A*)/*A*_0_] × 100.^36–39^ In this equation *D*, *A*_0_, and *A* are the degradation, absorbance at the beginning and time *t*, respectively.

Prior to irradiation, the suspension was shaken in the dark for 30 min to achieve the adsorption equilibrium of the organic dye on the DBNO NC surface. It is necessary to keep the photocatalyst in the dark before testing to achieve saturation (adsorption) and achieve a purely academic result. A good catalyst with no good adsorption for the substrate is not a useful system. Before the dye degradation experiment, the mixed photocatalyst and dye solution should be kept in the dark to reach the adsorption–desorption equilibrium. The equilibrium process itself is simply the reactants associating with the photocatalyst (absorption) and then diffusing away (desorption), this happens at a rate that is specific to the order of the system.

## 3. Results and discussion

In this study the sonochemical method was used for the synthesis of DBNO NC. Some of the determining parameters in this method are the frequency, power, amplitude, and sonication time. In this research, the effect of the sonication power on the purity and particle size of the nanocomposite were investigated. Ultrasound is employed as a swift tool for various tasks, such as architectural control of nanostructures. Cavitation created with the aid of ultrasound waves may result in favorable and specific nanoscale structures with a high uniformity. Owing to the hot-spot theory, the creation of excessively high temperatures and the release of immense amounts of energy can occur when bubbles collapse, these can be favorable to the conversion of massive structures to tiny particles. Thus, we can conclude that ultrasound irradiation can be very advantageous in the architectural control of nickelates.^40^ In the sonochemical method, the ˙H and ˙OH radicals are produced by adsorbing ultrasonic waves. The mechanism of this synthesis is related to the generation of radicals. In ultrasound synthesis, increasing the temperature and pressure inside the collapsing bubbles can cause the pyrolysis of water into ˙H and ˙OH radicals, and the following summarized mechanism is proposed:^41^Dy(NO_3_)_3_·5H_2_O + Ba(NO_3_)_2_ + Ni(NO_3_)_2_·4H_2_O + H_2_O → Dy^3+^ + Ba^2+^ + Ni^2+^ + 7NO_3_^−^
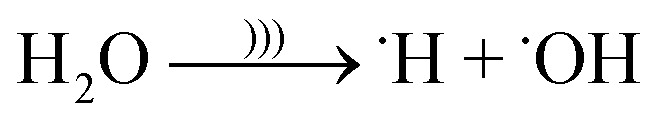
˙H + ˙H → H_2_˙OH + ˙OH → H_2_O_2_˙OH + other species → oxidized products

The hydroxyl radical is one of the strongest oxidants and can react non-selectively with almost all types of organic and inorganic compounds. The trapped organic compounds in the bubble either undergo pyrolysis or react with the hydroxyl radical. At the interface of the liquid–gas bubbles, the high temperature gradient leads to locally condensed ˙HO and the degradation reaction occurs in the aqueous phase. Although the temperature in this region is lower than that in the bubble core, there is an adequately high temperature for the thermal decomposition of the substrate. Moreover, H_2_O_2_ can be generated by the recombination of hydroxyl radicals during the sonication of a diluted aqueous solution, which does not usually play a crucial role in oxidizing organic species and the amount may be too small to be significant. Generally, there are two mechanisms responsible for the oxidation/degradation of pesticides by ultrasound, these are decided on the basis of the physical and chemical properties of the pesticides. The first mechanism is pyrolysis inside the cavitation bubbles, which is expected to be the main reaction path for the degradation of hydrophobic or apolar and more volatile compounds. The second mechanism is the formation of hydroxyl radicals in the cavitation bubbles, which are subsequently thrown out in the bulk liquid upon cavity collapse and oxidize the organic compounds, which are hydrophilic or polar and are non-volatile compounds. In the bulk liquid, the reactions are basically between the substrate and radicals that migrate from the interface. In the bulk phase, shear forces, turbulence and micro-streaming help the radical reaction to proceed more quickly. Most of the hydrophobic and volatile compounds react inside or at the interface of cavities, or inside the cavitation bubble, whereas hydrophilic and non-volatile compounds react in bulk water that contains insufficient ˙OH radicals.^42^

**Fig. 1:**
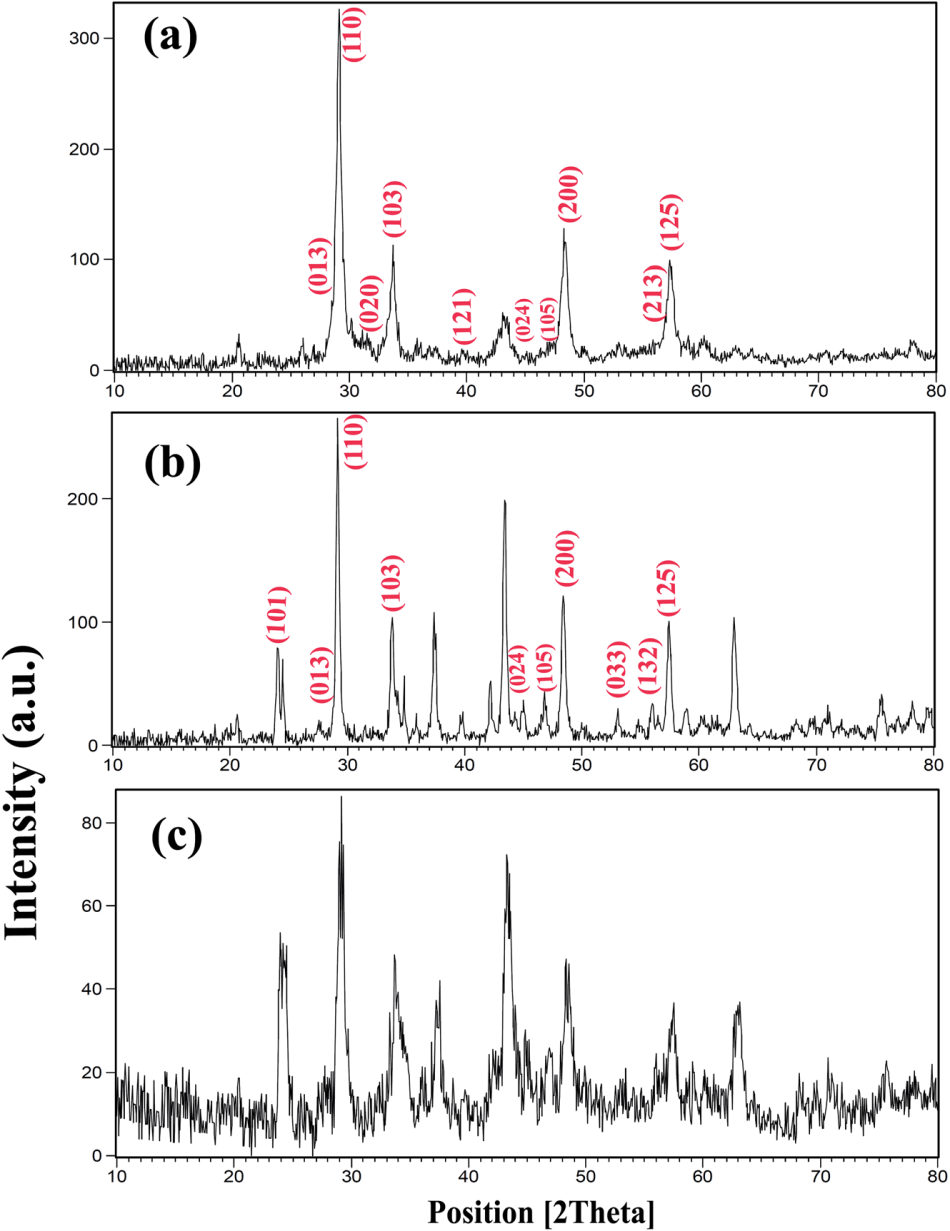
XRD patterns of samples prepared with different sonication powers: (a) 50 W (sample 3); (b) 30 W (sample 2); and (c) 15 W (sample 1).

Fig. 1 shows the X-ray diffractometry (XRD) patterns of samples prepared with different powers of ultrasound irradiation. In this work, three different powers, including 15, 30, and 50 W were used, as shown in Table 1. The concentration of the reagent, sonication, time and other conditions were kept constant in order to study this effect. This effect was investigated at room temperature. The peaks indexed in Fig. 1 correspond to BaDy_2_NiO_5_ (JCPDS no. = 00-041-0468, 01-088-1643). The pattern in Fig. 1a shows the formation of BaDy_2_NiO_5_ and Dy_2_O_3_ with a sonication power of 50 W. The prepared BaDy_2_NiO_5_ and Dy_2_O_3_ have orthorhombic and cubic systems. The products obtained with sonication powers of 30 and 15 W, are NiO, Dy_2_O_3_, and BaDy_2_NiO_5_, as shown in Fig. 1b and c, respectively.

**Fig. 2:**
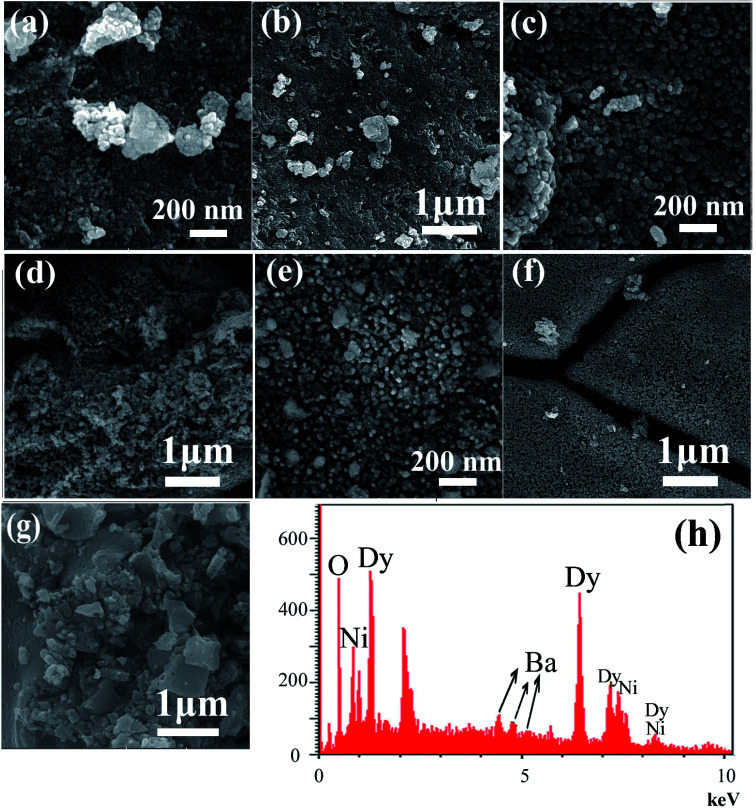
SEM images of DBNO NC prepared after 20 min sonication with different powers: (a) and (b) 15 W (sample 1); (c) and (d) 30 W (sample 2); and (e) and (f) 50 W (sample 3). (g) The blank sample and (h) the EDS spectrum of sample 2.

The effect of the sonication power on the morphology and particle size of the nanocomposite was investigated. The SEM images shown in Fig. 2 show the formation of agglomerated nanoparticles in all three samples synthesized with sonication powers of 15, 30, and 50 W. The images show that with the increasing sonication power from 15 W (sample 1, Fig. 2a and b) to 30 W (sample 2, Fig. 2c and d), and then 50 W (sample 3, Fig. 2e and f), the particle size is decreased. The high ultrasound power can increase the nucleation rate, thus the number of nucleation centers increases and the agglomeration decreases. This fact leads to the formation of nanoparticles with small particle sizes, if the sonication power is high. The ultrasonic waves can modify the morphology of the products and decrease the super saturation limits. Thus, the particle size is decreased by increasing the ultrasonic power.^43^ In addition, a blank sample was prepared in the absence of ultrasound irradiation. SEM images of this sample showed the formation of aggregated and bulk structures, as shown in Fig. 2g. These results confirm the importance of the presence of ultrasound irradiation for the synthesis of nanomaterials. Fig. 2h shows an EDS spectrum of sample 2, prepared with a sonication power of 30 W. The spectrum indicates the presence of O, Ni, Ba, and Dy elements. The EDS results confirm the XRD results (Fig. 1b).

**Fig. 3:**
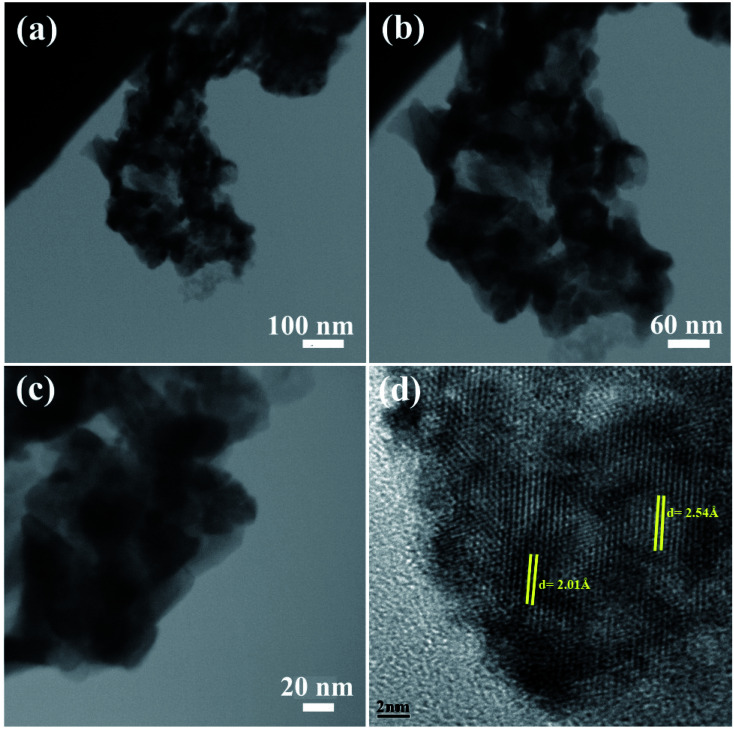
TEM images of sample 2.

The morphology of the NC was also studied using TEM images. Fig. 3 shows the TEM images of sample 2. These images show the formation of the agglomerated nanoparticles with an average particle size of approximately 50 nm. The TEM images confirm the SEM results shown in Fig. 3a–c. The HRTEM image of the BaDy_2_NiO_5_ nanocomposite is displayed in Fig. 3d. The crystalline planes recognized by the parallel lines indicate the high degree of crystallinity of the nanocomposite. The lattice fringes are clearly shown with spacing fringes of 2.01 and 2.54 Å, which match well with the crystal planes (200) and (122) of cubic NiO and the orthorhombic BaDy_2_NiO_5_ crystals, respectively.

**Fig. 4:**
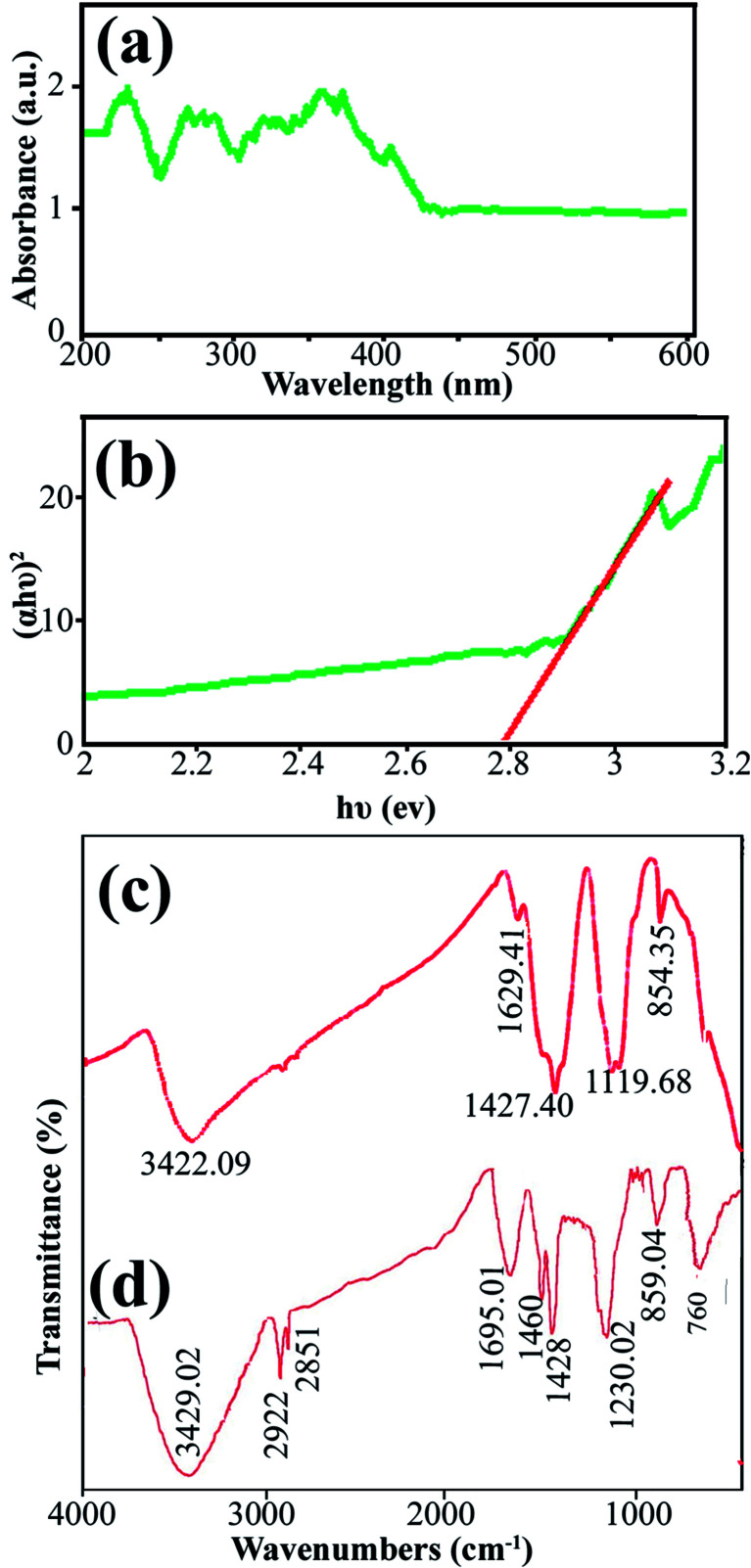
(a) and (b) DRS results for the product obtained with a sonication power of 30 W (sample 2), and (c) and (d) the FT-IR results for sample 2 and the core almond.

Data on the surface coordination and electronic states of the compound obtained by measuring the d–d and f–d electron transitions, and also the oxygen metal ion charge transfer, can be obtained from the DRS measurement. DRS was used to investigate the semiconductor behavior of DBNO NC. Fig. 4a shows the DRS spectrum of sample 2. The sample shows absorption bonds at 200–420 nm. The curve of (*αhυ*)^2^*versus hυ*, shown in Fig. 4b, indicates that the band gap of DBNO NC (sample 2) is 2.77 eV.

In order to clarify the functional groups on the surface of the nanocomposites, the FT-IR spectra of sample 2 and the core almond were recorded and are shown in Fig. 4c and d, respectively. The significant absorption bands at 3422.09 and 3429 cm^−1^ in the spectra of sample 2 and the almond are related to the O–H stretching absorption bands and are due to the presence of OH functional groups such as alcohols, phenols and carboxylic acids.^36,39^ In Fig. 4d, the absorption bands at 2922 and 1695.01 cm^−1^ are related to the C–H and C=O stretching vibrations, respectively. The absorption bands at 1460 and 1428 cm^−1^ indicate the presence of aromatic C=C stretching. In addition, the band at 1230.02 cm^−1^ represents the C–O stretching vibration. The absorption bands in 859.04 and 760 cm^−1^ are caused by the C–H out of plane bending and O–H out of plane bending vibrations, respectively. Fig. 4c and d indicate that the almond plays an important role as a reducing and capping agent owing to the biomolecules capping on the surface of the NCs. On the other hand, there is a difference in the shape and shift of the absorption bands owing to the interaction between the cations and active sites of the almond. In Fig. 4c, the absorption bands related to the C=O and C–O stretching vibrations in DBNO NC have been shifted to lower wavenumbers, 1629 and 1119 cm^−1^, respectively, compared to the almond. These bands revealed the functional groups responsible for the stabilization.^44^ The peak at about 500 cm^−1^ in Fig. 4c is related to the M–O tetragonal/octahedral vibrations (M = metal).^37,38^

**Fig. 5:**
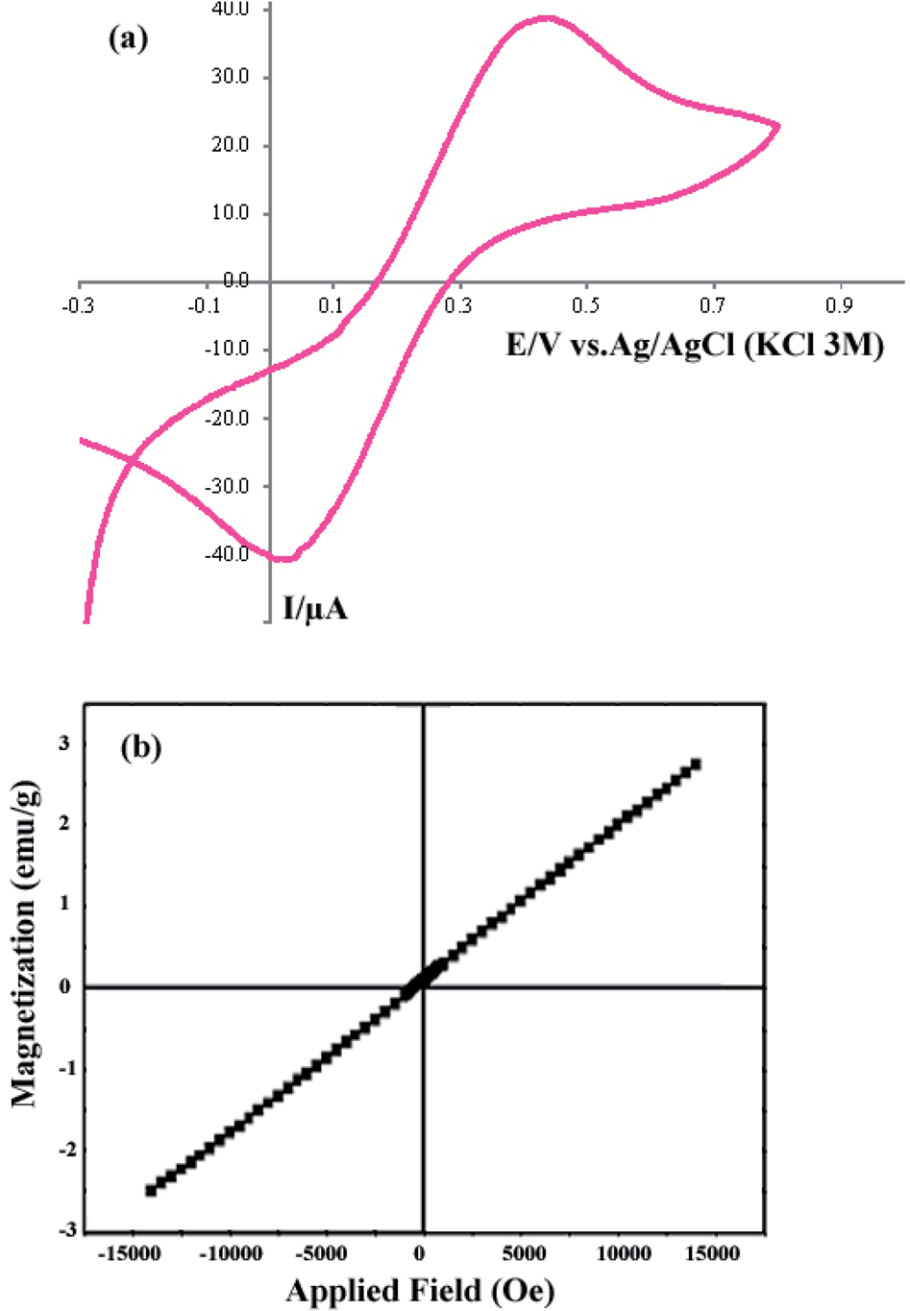
(a) Cyclic voltagram of the product obtained with a sonication power of 30 W (sample 2), and (b) VSM curve of sample 2.

The CV curve of sample 2 in the potential range −0.3 to +0.8 V is shown in Fig. 5a. For the CV analysis, three electrodes including working, counter, and reference electrodes were used. In this work, Ag/AgCl (3.0 M KCl) was used as the reference electrode, and Pt and a glassy carbon electrode (GCE) were used as the counter and working electrodes, respectively. The scan rate was selected as 0.1 V s^−1^. Fig. 5a shows that the cathodic current and voltage are −40.72 μA and 0.011 V, respectively. Furthermore, the anodic current and voltage are +38.748 μA and 0.436 V, respectively. The CV curve shows the electrochemical behavior of the DBNO NC.

The VSM curve shown in Fig. 5b shows the magnetic behavior of DBNO NC. The linear variation of this curve indicates the AFM behavior for the product obtained with a sonication power of 30 W (sample 2). The reported AFM behavior is similar to that observed in previous studies. Upadhyay, Garcia-Matres, and Basu *et al.* demonstrated this behavior for Tb_2_BaNiO_5_, Dy_2_BaNiO_5_, and Er_2_BaNiO_5_ nickelates, respectively.^10,15,18^

**Fig. 6:**
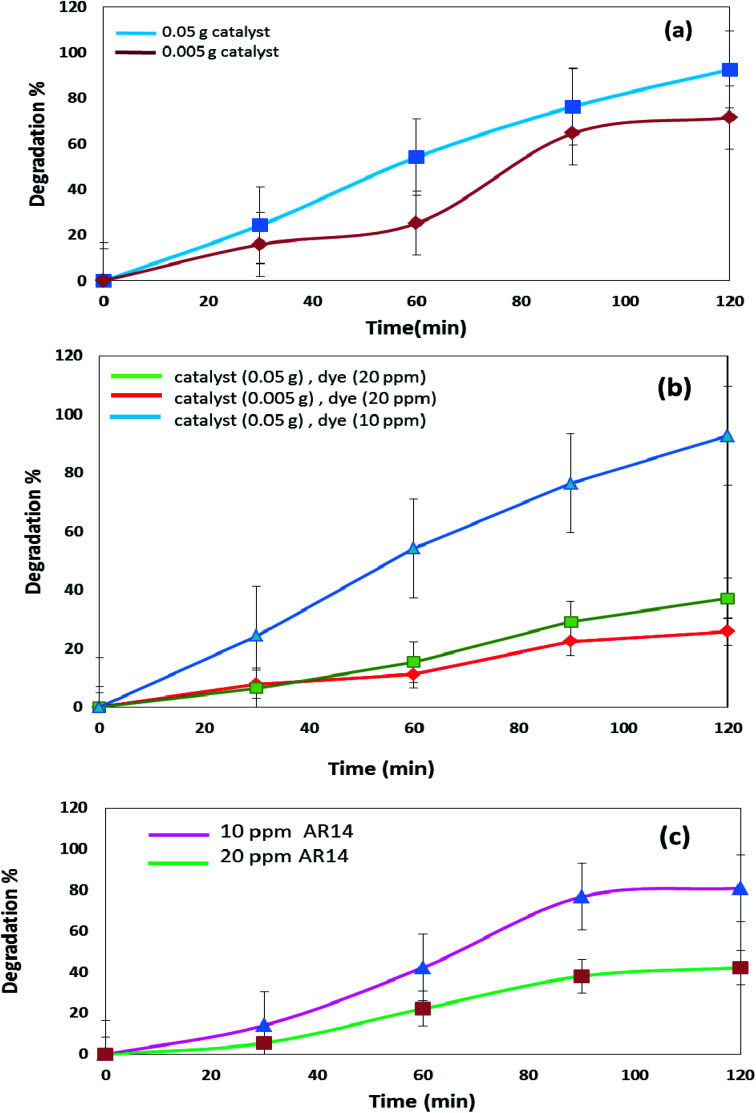
(a) Photocatalytic degradation of AB92 in the presence of different amounts of catalyst, under UV light irradiation, and (b) and (c) effects of the concentration of dye in the photocatalytic degradation of AB92 and AR14, respectively, under UV light irradiation.

**Fig. 7:**
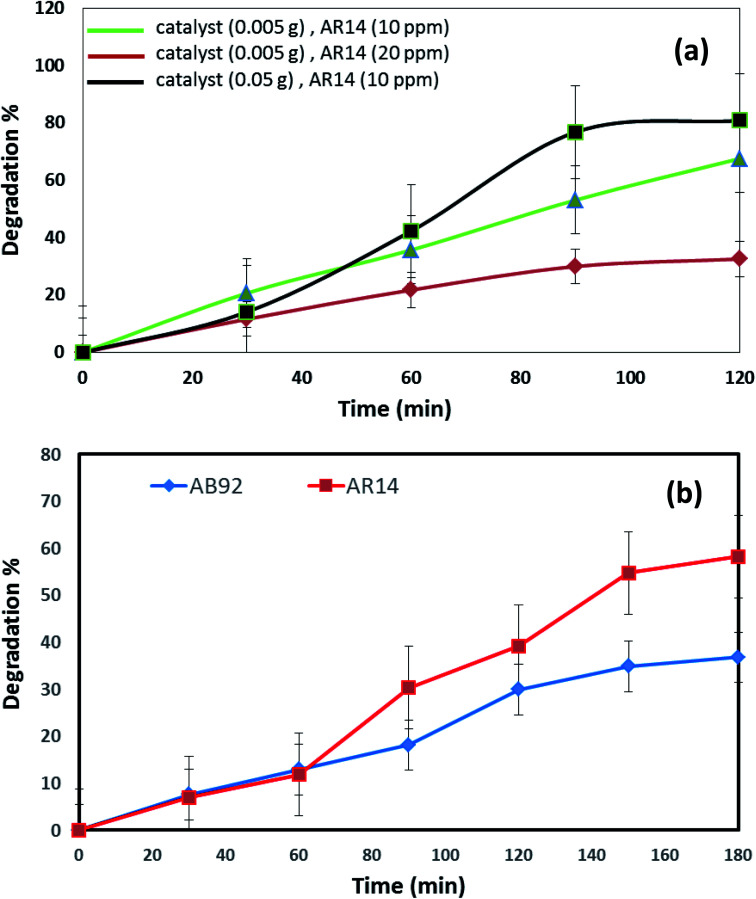
(a) Effects of the concentration of dye and amount of catalyst in the photocatalytic degradation of AR14 under UV light irradiation, and (b) comparison of the photocatalytic activity of DBNO NC for the degradation of AB92 and AR14 in the presence of Vis light.

The DBNO NC synthesized in this work were used as photocatalysts. Fig. 6 and 7 confirm the photocatalytic activities of these nanocomposites for the degradation of AB92 and AR14 dyes. These dyes are present in water as organic pollutants and pose a serious water pollution threat to human societies. The treatment of wastewater containing AB 92 and AR14 is a challenge. Thus, we have selected these two anionic dyes and investigated the photocatalytic activity of DBNO NC for the degradation of these two dyes. In this study, we investigated the effects of the amount of photocatalyst, the concentration of dye solution, the type of organic dye, and the type of light irradiation on the photocatalytic activity of the as-prepared nanocomposite. The results have been summarized in Scheme 1. The effect of the amount of DBNO NC, as the photocatalyst, on the degradation of AB92 under UV light irradiation, has been studied in Fig. 6a and b. Fig. 6a shows the investigation of this effect on the degradation of a 10 ppm solution of AB92. In this study, two different amounts of the catalyst, 0.05 and 0.005 g, were used. Fig. 6a shows the photocatalytic degradation of AB92 in the presence of 0.005 and 0.05 g of catalyst was 71.47% and 92.58%, after 120 min, respectively. This figure shows that with the increasing amount of catalyst, the percentage of photocatalytic degradation of the dye increased. The enhancement of the surface area, and thus, the improvement in the absorption of AB92 on the surface of the DBNO NC, may be the reason for the increment in the decomposition yield.^40^

Two solutions with different concentrations of AB92, 10 and 20 ppm, were prepared. The degradation of these solutions in the presence of 0.05 g of catalyst under UV light irradiation was investigated. Fig. 6b shows that the degradation of AB92 decreased from 92.59% to 37.10%, after 120 min, with the increasing concentration of dye from 10 to 20 ppm, respectively. The saturation of the DBNO NC layers and surfaces with the increasing concentration of dye solution may be the reason for the decrement of the catalytic yield. Fig. 6b also confirms the results of Fig. 6a and shows that with an increasing amount of catalyst, the percentage of photocatalytic degradation of the dye increased.

The effect of the dye concentration was also studied in the photocatalytic degradation of AR14 under UV light irradiation. Fig. 6c shows that upon decreasing the AR14 concentration from 20 to 10 ppm, the photocatalytic degradation is increased from 42.22% to 80.83%, respectively. This study investigated the use of 0.05 g of DBNO NC as a photocatalyst. The results obtained from the investigation of the effect of the dye concentration on the photocatalytic degradation of both the AB92 and AR14 dyes are the same, as shown in Fig. 6b and c.

The effects of the dye concentration and the amount of catalyst in the photocatalytic degradation of AR14 are investigated under UV light irradiation, as shown in Fig. 7a. This figure indicates that with the increasing amount of catalyst and the decreasing concentration of AR14, the photocatalytic activity is increased. The obtained results are similar to those obtained in the photocatalytic degradation of AB92 (Fig. 6a and b). Table 2 shows the amount of adsorption of the anionic dyes on the sample after 30 min.

**Table 2:** The amount of adsorption of the anionic dyes on the sample after 30 min

Anionic dye	Amount of dye	Time	Catalyst (0.005 g)	Adsorption of dye on the sample
AB92	10 ppm	30 min	Dy_2_BaNiO_5_ NC	0.716
AB92	20 ppm	30 min	Dy_2_BaNiO_5_ NC	7.89
AR14	10 ppm	30 min	Dy_2_BaNiO_5_ NC	1.245

The photocatalytic activity of DBNO NC for the degradation of AB92 and AR14 in the presence of Vis light is compared in Fig. 7b. In this comparison, 0.005 g of catalyst for the degradation of a dye solution of 10 ppm was used. Fig. 7b shows that in the presence of Vis light and 0.005 g of DBNO NC, the degradation percentages of AR14 and AB92 after 180 min are 58.25% and 36.79%, respectively. Thus, in the presence of Vis light, the percentage of the photocatalytic degradation of AR14 is higher than that of AB92. The hydroxyl radicals, generated *via* the reaction of the hydroxide ions (or water molecule) with the positive pores of the catalyst surface, have a determining role in the decolorization process.^37^ In the presence of an anionic dye (AB92), owing to the presence of a negative charge, the adsorption of the dye on the surface of the DBNO, with a high surface electron density, is reduced and consequently the concentration of the hydroxyl radicals is reduced. In these conditions, the decolorization efficiency is finally reduced. On the basis of the above-mentioned observations, in the presence of AR14 as an inert dye, more hydroxyl radicals are created compared to the number created in the presence of AB92. Thus, the degradation of AR14 is higher than AB92 in the presence of the as-prepared photocatalysts in this work. We performed more photocatalytic tests to investigate the effect of the dye type on the photocatalytic activity. Only one parameter was changed in each experiment, the other parameters remained constant.

Comparison of the photocatalytic degradations of AR14 and AB92 in different conditions under UV light irradiation (Fig. 6 and 7) shows that in high concentrations of dye solution, the degradation of AR14 is higher than that of AB92. These results are similar to those obtained in Vis light. However, in lower concentrations, the results obtained oppose those reported in high concentrations. The reduced degradation of AB92 compared to AR14 in high concentrations of the dye solution may be related to its larger molecular size. It is very important to note that the photodegradation of the dye depends not only on the formation of hydroxyl radicals at the interface of the nanocomposite, but also on the ability of the dye to be adsorbed on the surface of the photocatalyst. Furthermore, as the physical and chemical properties of different dyes are not the same, two dyes compete for adsorption at the surface of the photocatalyst.^37^

**Fig. 8:**
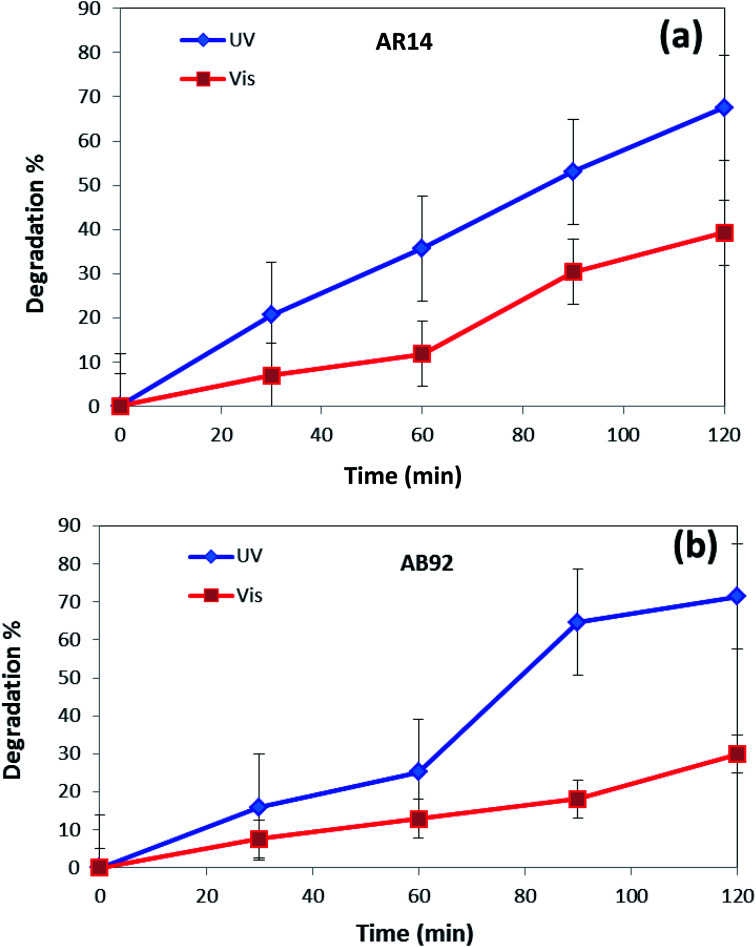
Comparison of the photocatalytic activity of DBNO NC under UV and Vis irradiations, for the degradation of: (a) AR14; and (b) AB92.

In Fig. 8, the photocatalytic degradation of AR14 and AB92 under UV and Vis irradiation is compared. The figure shows the higher photocatalytic degradation of both dyes under UV irradiation. This can be ascribed to the separation of the photogenerated electron–hole pairs and the desired absorption ability of the nanocomposite under UV irradiation. The presence of significant absorption bands in the UV region in the DRS spectrum of the DBNO NC (Fig. 4a) confirms the greater photocatalytic efficiency of the nanocomposites under UV irradiation.

The photocatalytic mechanism for the removal of the AB92/AR14 molecules can be denoted as follows:^45–47^DBNO NC + *hυ* → DBNO NC* (h_VB_^+^ + e_CB_^−^)DBNO NC (e_CB_^−^) + O_2_ → DBNO NC + O_2_˙^−^DBNO NC (h_VB_^+^) + H_2_O → DBNO NC + H^+^ + OH˙O_2_˙^−^ + H^+^ → HO_2_˙^−^AB92/AR14 + OH˙ → degradation productsAB92/AR14 + O_2_˙^−^ → degradation productsAB92/AR14 + h_VB_^+^ → oxidation productsAB92/AR14 + e_CB_^−^ → reduction products

## 4. Conclusions

The present work reports the ultrasonic-assisted green synthesis of DBNO NC in the presence of core almond as a capping agent. XRD analysis showed that the product is a BaDy_2_NiO_5_ nanocomposite. The morphologies of the products were obtained using SEM. The SEM images showed the formation of agglomerated nanoparticles in all samples synthesized with different powers of sonication. With increasing sonication power, the particle size decreased. TEM images were obtained and the results are in good agreement with the SEM results. The optical, electrical, magnetic, and photocatalytic properties of the nanocomposite were investigated. The bandgap of DBNO NC was obtained at 2.77 eV. The nanocomposite prepared in this work shows AFM behavior and can be used as a photocatalyst. The results of the catalytic surveys demonstrate that DBNO NC can be utilized as a helpful and substantial photocatalyst for cleaning water, as well as environmental remediation. The effects of the amount of photocatalyst, concentration of dye solution, type of organic dye, and also light irradiation on the photocatalytic activity of DBNO NC were studied. Upon increasing the amount of photocatalyst and also decreasing the dye concentration, the photocatalytic activity of DBNO NC is increased.

## Conflicts of interest

The authors declare that there are no conflicts of interest regarding the publication of this manuscript.

## Supplementary Material
